# Validation of the German version of the Nurse-Work Instability Scale: baseline survey findings of a prospective study of a cohort of geriatric care workers

**DOI:** 10.1186/1745-6673-8-33

**Published:** 2013-12-13

**Authors:** Melanie Harling, Anja Schablon, Albert Nienhaus

**Affiliations:** 1 University Medical Center Hamburg-Eppendorf Competence Centre for Epidemiology and Health Services Research in Nursing (CVcare), Martinistr. 52, Building O17, 20246 Hamburg, Germany; 2 Department of Occupational Health Research, Institution for Statutory Accident Insurance and Prevention in the Health and Welfare, Services, Pappelallee 35/37, 22089 Hamburg, Germany

**Keywords:** Nurse-work instability scale, Nurses, Musculoskeletal disorders

## Abstract

**Background:**

A prospective study of a cohort of nursing staff from nursing homes was undertaken to validate the Nurse-Work Instability Scale (Nurse-WIS). Baseline investigation data was used to test reliability, construct validity and criterion validity.

**Method:**

A survey of nursing staff from nursing homes was conducted using a questionnaire containing the Nurse-WIS along with other survey instruments (including SF-12, WAI, SPE). The self-reported number of days’ sick leave taken and if a pension for reduced work capacity was drawn were recorded. The reliability of the scale was checked by item difficulty (P), item discrimination (r_jt_) and by internal consistency according to Cronbach’s coefficient. The hypotheses for checking construct validity were tested on the basis of correlations. Pearson’s chi-square was used to test concurrent criterion validity; discriminant validity was tested by means of binary logistic regression.

**Results:**

396 persons answered the questionnaire (21.3% response rate). More than 80% were female and mostly work full-time in a rotating shift pattern. Following the test for item discrimination, two items were removed from the Nurse-WIS test. According to Cronbach’s (0.927) the scale provides a high degree of measuring accuracy. All hypotheses and assumptions used to test validity were confirmed: As the Nurse-WIS risk increases, health-related quality of life, work ability and job satisfaction decline. Depressive symptoms and a poor subjective prognosis of earning capacity are also more frequent. Musculoskeletal disorders and impairments of psychological well-being are more frequent. Age also influences the Nurse-WIS result. While 12.0% of those below the age of 35 had an increased risk, the figure for those aged over 55 was 50%.

**Conclusion:**

This study is the first validation study of the Nurse-WIS to date. The Nurse-WIS shows good reliability, good validity and a good level of measuring accuracy. It appears to be suitable for recording prevention and rehabilitation needs among health care workers. If, in the follow-up, the Nurse-WIS likewise proves to be a reliable screening instrument with good predictive validity, it could ensure that suitable action is taken at an early stage, thereby helping to counteract early retirement and the anticipated shortage of health care workers.

## Background

Demographic trends in Germany are expected to lead to a substantial increase in the number of people needing care, and therefore to a requirement for 500,000 additional health care workers [1-4].

Among other things, this means that we need to keep health care workers healthy and motivated to work until retirement age. However, health care work involves considerable strains that can present a challenge, especially for employees over 50 years of age. The literature shows that the work ability of nurses and geriatric nurses declines with increasing age [5] and that cervical spine and lumbar spine problems become more common [6-8]. Moreover, health care workers have an increased risk of developing Musculoskeletal disorders (MSDs) [9-13]. Transferring patients involves moving heavy load weights [14-17]. Additional risk factors are frequent bending and twisting of the torso, along with static body postures [18-20]. The literature shows back problem prevalence rates of 30% to 60% among health care workers [21-23]. Burnout, psychological impairments, low job satisfaction and poor general health are also common in care work [6,24-29].

MSDs and psychiatric disorders such as depression are also the most frequent reasons for long-term sick leave, and employees above the age of 50 are more likely to be affected [30].

Moreover, long-term sick leave due to such disorders is often a transitional stage on the way to reduced work capacity or work disability [31]. In Germany, if a major health impairment makes it no longer possible to work, or possible only to a limited extent, a pension for reduced work capacity is paid. Psychiatric disorders, MSDs and cancer are the most common grounds for payment of a pension for reduced work capacity [32].

Harling *et al*. [33] ascertained that health care workers more frequently draw a pension for reduced work capacity than other occupational groups. Additionally, MSDs are more often the reason for rehabilitation among health care workers, and after rehabilitation the risk of a pension for reduced work capacity was higher than for other occupational groups.

If the proportion of health care workers drawing a pension for reduced work capacity increases, the strained situation in the geriatric and health care sector could be further aggravated.

In efforts to counteract the forecast shortage of health care workers, maintaining their work ability will be a central concern. Many studies have shown the effectiveness of interventions targeted at individuals with onset symptoms of MSDs [34-36] or individuals who run an increased risk of reduced work ability [37]. Offers of this kind could also be useful for maintaining health care workers’ capacity to work. Until now there has been a lack of effective screening instruments to facilitate the offer of early interventions for health care workers at risk. A new questionnaire that appears to meet these requirements is the Nurse-Work Instability Scale (Nurse-WIS) [38]. Until now, however, the questionnaire was only available in English. It was therefore translated into German and this version of the Nurse-WIS was validated in a prospective study of a cohort of nursing staff from nursing homes. The results of the baseline investigation, which tested reliability, construct validity and criterion validity, are described below.

## Methods

### The Nurse-Work Instability scale (Nurse-WIS)

The concept of work instability was developed at the University of Leeds and has been defined as follows: “*Work Instability* (*WI*) *has been defined as a state in which the consequences of a mismatch between an individual*’*s functional and cognitive abilities and the demands of his or her job can threaten continuing employment if not resolved*” [39].

The concept is based on the premise that there is often a period before work disability when there is difficulty in fulfilling work tasks. Interventions at this point in time may prevent the impending loss of work capacity. Consequently, early identification of work instability is the key to preventing long-term sick leave or reduced work capacity. The concept of work instability has already been explored for various clinical fields such as rheumatoid arthritis [39], ankylosing spondylitis (Bechterew’s disease) [40] and for post-traumatic intracranial injuries [41].

The Nurse-WIS is an occupation-specific instrument for recording health care workers who have difficulties in performing their work. It was developed from qualitative interviews with health care workers and it covers all areas that are important to them. Along with musculoskeletal complaints, it records psychosocial factors. The scale comprises 30 items that can be answered by 1 = ‘true’ and 0 = ‘false’. The points for all the answers are added up to calculate the total score. The higher the total score, the higher the risk of work instability. A score of < 10 points signifies a low risk, 10–19 points a moderate risk and a figure of ≥ 20 points an increased risk of work instability [38].

The Nurse-WIS was translated into German with the help of a ‘forward-backward procedure’ [42]. First, the original English version was translated into German. This version was then retranslated into English. A workshop of experts (one occupational health specialist, one epidemiologist, one professor of nursing science, two health care researchers, one psychologist) compared and discussed the original English version, the German version and the retranslated version and reached a verdict by committee assessment. This version was tested in a pre-test with n = 87, with no significant changes occurring.

### Study design, recruitment of participants and data protection

In order to test the reliability, validity and forecasting capabilities of the Nurse-WIS, nursing staff from nursing homes took part in a prospective cohort study. The baseline investigation involved surveying the study participants on the basis of a standard questionnaire (end of 2010). The follow-up took place one year later. Using the data obtained from the baseline investigation, we tested the reliability and validity of the Nurse-WIS and the results are described below. The plan is to use data from the follow-up to test the predictive validity of the Nurse-WIS. This data is currently being analysed and prepared for publication.

Study participants were recruited via geriatric nursing homes. The nursing homes were selected by taking a random sample of member companies of our cooperation partner, the German Institution for Statutory Accident Insurance and Prevention in the Health and Welfare Services (BGW). For randomization a random sample of the original list of member companies was drawn with SPSS. The BGW contacted the nursing homes from the random sample by telephone to inform them. Those that agreed to the study were sent the documentation, which they displayed in the workplace for their staff. A total of 83 nursing homes were contacted, of which 29 (34.9%) were willing to take part in the study. Each set of study documents was packed individually in blank form in an A4 envelope. Each envelope contained a letter of information about the study and data protection, a declaration of consent, the questionnaire and a stamped addressed envelope for return. The envelope was addressed to the University Medical Center Hamburg-Eppendorf (UKE), Competence Centre for Epidemiology and Health Services Research in Nursing (CVcare), which undertook the analysis. The health care workers could fill in the study documents either at their workplace or at home before returning them. In this way, the nursing home as employer neither knew which health care workers had taken part in the study, nor saw the documents after they had been filled in. This procedure was developed with the help of the Hamburg Data Protection Commissioner. The study was conducted following the requirements of the Helsinki Declaration and the ethics commission of the Hamburg Medical Association also gave a positive verdict on the conduct of the study (reference number PV3463). A pre-test (with a 37% response rate) was carried out from May to June 2010.

### The survey instrument, criteria for inclusion and exclusion

The questionnaire was used to record socio-demographic features (gender, age, country of origin, education) and occupational data (length of service, scope of employment [e.g. full-time], rotating shifts, etc.). Two questions were used to record applications for a pension for reduced work capacity (‘Are you currently thinking about applying for a pension [early retirement pension on health grounds or pension for reduced work capacity]?’ and ‘Have you already applied for a pension?’).

Along with the Nurse-WIS, we used other validated and tested scales, as follows:

•Work Ability Index (WAI) [43,44].

•Short Form Health Survey (SF-12) [45,46].

•Subjective Prognosis of Work Capacity (SPE Scale) [47,48].

•Job satisfaction scale from the German version of the Copenhagen Psychosocial Questionnaire (COPSOQ) [49,50].

•German version of the Center for Epidemiological Studies Depression Scale/CES-D-Scale [51].

We recorded the number of days’ sick leave during the previous 12 months, along with the reason for each period of absence. This information was used to form the following central study variables:

•Total number of days’ sick leave during the previous 12 months.

•Long-term sick leave during the previous 12 months (> 42 days per period of absence).

In Germany, long-term sick leave is taken to mean an absence due to sickness of > 42 days, because after that the payment of salary by the employer is replaced by sick pay from statutory health insurance providers [30].

•Work-related MSD.

There is currently no standard definition of which disorders count as work-related MSD. The literature regards disorders affecting the back and the upper extremity as work-related MSD [52-54].

•Impairments of psychological well-being.

Conditions such as ‘burnout’, ‘total exhaustion’ or ‘depressive malaise’ mentioned by study participants as reasons for sick leave were summarised under this heading.

•Other disorders.

All other disorders, such as acute respiratory infections, gastro-intestinal illnesses, degenerative diseases and injuries were summarised under this heading.

Since the number of days’ sick leave and the reason for each period of absence are central variables for the purpose of analysis, individuals who provided no information on these items were excluded from the study. Persons who were not employed as geriatric care workers (e.g. voluntary helpers) were also excluded.

### Testing reliability

We observed the psychometric item difficulty (P), the item discrimination (r_jt_) and the internal consistency on the basis of Cronbach’s coefficient. *P* describes the percentage of persons who marked the answer ‘true’ in the Nurse-WIS. Items with an extremely low or high *P* cannot show up differences between individuals [55]. For example, if 99% (P = 0.99) of all persons marked one Item of the Nurse-WIS with ‘true’, it can be assumed that this item is not adequate. Items with P < 0.1 or P > 0.9 were therefore excluded. In the case of *r*_*jt*_, as high a figure as possible is desirable. Items with negative discrimination are unsuitable for the scale [55,56]. Items with a discrimination *r*_*jt*_ < 0.3 were therefore excluded.

The coefficient can take values between minus infinity and 1, with reliabilities between ≥ 0.8 and ≤ 0.9 regarded as being moderate and reliabilities of > 0.9 as high [56].

### Testing construct validity

The construct validity of a test, given as a correlation coefficient, reflects the extent to which the construct that is to be measured is related to other variables that it should theoretically be associated with. Correlation coefficients between 0.4 and 0.6 are classified as moderate validity and coefficients of more than 0.6 as high validity [56]. For testing construct validity, we took the total score of the scale as the result of the Nurse-WIS. The hypotheses for testing construct validity are:

•‘With an increased risk according to the Nurse-WIS, health-related quality of life (SF-12) declines’.

•‘With an increased risk according to the Nurse-WIS, the Work Ability Index (WAI) declines’.

•‘With an increased risk according to the Nurse-WIS, job satisfaction (COPSOQ) is low’.

•‘With an increased risk according to the Nurse-WIS, the probability of depressive malaise (ADS) increases’.

•‘With an increased risk according to the Nurse-WIS, capacity to work is subjectively (SPE) assessed as at risk’.

### Testing concurrent criterion validity

Criterion validity exists if the result from a scale for measuring a latent variable matches the results for a corresponding manifest criterion [56]. As a manifest criterion we used the variable concerning the existence of certain diseases. The risk categories of the Nurse-WIS were dichotomised so as to produce a new variable with the characteristics ‘low/moderate risk’ and ‘high risk’. We used Pearson’s chi-square to test whether the criteria correlated with the result of the Nurse-WIS. The underlying assumptions and hypotheses for testing concurrent criterion validity are described below.

•Work-related musculoskeletal disorders.

Sick leave in the previous year due to a musculoskeletal disorder is a predictor for long-term sick leave in the subsequent year [30,57]. Persons with an MSD are therefore expected to more frequently show an increased risk according to the Nurse-WIS.

•Psychological impairments to well-being.

Psychosocial factors frequently correlate with the emergence and chronification of musculoskeletal disorders (MSD) [58-61]. MSD are also associated with burnout and depression [61,62]. Impairments of psychological well-being in the previous year are also predictors for long-term sick leave in the subsequent year [30,63]. Persons with impairments of psychological well-being are therefore expected to more frequently indicate an increased risk in accordance with the Nurse-WIS scale.

### Testing discriminant criterion validity

Discriminant criterion validity examines if the relationship between the criterion and the result of the scale differs in different populations [56]. Since the frequency of MSD increases with age [30,31], one can assume that MSD are more common among older health care workers. Age could therefore act as a moderating variable. Likewise, gender, educational level, the type of nursing training and length of service could also act as moderating variables. However, if the Nurse-WIS is a suitable instrument, the result of the scale, regardless of said moderating variables, can be expected to correlate with the criteria (musculoskeletal disorder, psychological impairment to well-being) for testing concurrent criterion validity.

Binary logistic regression was used to test the influence of moderating variables. To create a model, we used the ‘stepwise downwards’ methods of Hosmer & Lemeshow [64]. The final model includes the variables that have an influence on the target variable (risk according to the Nurse-WIS).

## Findings

### Description of the study population

Of 1,816 questionnaires sent to 29 geriatric nursing homes, 396 (21.3%) were returned fully completed by respondents who met the criteria for inclusion. More than 80% of the study participants were female and the majority were aged between 36 and 45 or between 46 and 55. More than half had a secondary school certificate. More than 60% had completed a three-year training course in geriatric care or nursing. As regards length of service, 44.4% of study participants had worked from 0 to 10 years in the health care sector, 30.6% for 11 to 20 years, 14.6% for 21 to 30 years and more than 10% had been health care workers for more than 30 years. The majority of study participants worked full time and most worked in a rotating shift pattern but not at night (Table 1).

**Table 1:** Description of the study population

	**n = 396 % (n)**
**Gender**	
Female	82.6% (327)
Male	17.4% (69)
**Age**	
17 to 35 years	37.9% (150)
36 to 45 years	26.8% (106)
46 to 55 years	26.3% (104)
Over 55	9.1% (36)
**Grew up in**	
Germany	86.6% (343)
Other countries	13.4% (53)
**Education**	
Lower secondary, elementary school certificate	28.5% (113)
Secondary school certificate	53.3% (211)
High school/university entrance certificate	18.2% (72)
**Vocational training**	
Qualified geriatric nurse or nurse	61.9% (245)
Geriatric care or nursing assistant	23.7% (94)
Employee without nursing training^1^	14.4% (57)
**Length of service**	
0–10 years	44.4% (176)
11–20 years	30.6% (121)
21–30 years	14.6% (58)
More than 30 years	10.4% (41)
**Scope of employment**	
Full time (≥ 35 hours a week)	68.9% (273)
Part time (15–34 hours a week)	29.3% (116)
Part time (< 15 hours a week)	1.8% (7)
**Working hours**	
Rotating shifts excluding nights	56.6% (224)
Rotating shifts including nights	26.3% (104)
Day duty, always at the same times	9.8% (39)
Only night work	7.3% (29)

^1^and trainees, individuals doing civilian national service and persons undertaking a voluntary year of social service.

### Sick leave, pensions for reduced work capacity and Nurse-WIS

Around 20% had taken sick leave because of a musculoskeletal disorder (MSD) during the previous 12 months, and 6.3% because of a psychological impairment to well-being. The most common reason (44.9%) for an absence due to sickness was other illnesses (e.g. acute respiratory infection). The percentage that had taken long-term sick leave due to an MSD was 2.5%, while the figure for long-term sick leave due to impairments of psychological well-being was 1.3%. Two individuals (0.5%) said they had applied for early retirement or for a pension for reduced work capacity. The median total score on the Nurse-WIS was 13.1 points. The Nurse-WIS showed 35.1% as having a low risk, 41.2% a moderate risk and 23.7% an increased risk (Table 2).

**Table 2:** Sick leave, applications for pensions due to reduced work capacity and results of the Nurse-WIS

	**n = 396 % (n)**
**Sick leave:**	
**Musculoskeletal disorders**	
No	79.5% (315)
Yes	20.5% (81)
**Psychological impairments to well-being**	
No	93.7% (371)
Yes	6.3% (25)
**Degenerative disease/disease of the lower limbs**	
No	94.2% (373)
Yes	5.8% (23)
**Other illnesses**	
No	44.9% (178)
Yes	55.1% (218)
**Long-term sick leave (> 42 days):**	
**Long-term sick leave because of MSD**	
No	97.5% (386)
Yes	2.5% (10)
**Long-term sick leave because of impairments of psychological well-being**	
No	98.7% (391)
Yes	1.3% (5)
**Pensions for reduced work capacity:**	
**Application for pension for reduced work capacity**	
No	99.5% (394)
Yes	0.5% (2)
**Nurse-WIS**	
**Total score**	
Median (IQR)	13.1 pts (12.0 pts)
Minimum/maximum	0.0 pts/28.0 pts
**Risk categories**	% (**n**)
Low risk (< 10 points)	35.1% (139)
Moderate risk (10–19 points)	41.2% (163)
Increased risk (≥ 20 points)	23.7% (94)

*IQR* = interquartile range.

### Reliability of the Nurse-WIS

The difficulty index for all items is within an acceptable range. In the case of item discrimination, a coefficient of *r*_*jt*_ < 3 is stated for two items. These items are therefore not suitable for recording the risk of work instability in accordance with the Nurse-WIS and were excluded from the analysis. The total score of the Nurse-WIS was calculated on the basis of the remaining 28 items. Cronbach’s coefficient is 0.927. Consequently, the scale with 28 items is highly reliable (no table).

### Construct validity of the Nurse-WIS

**Figure 1:**
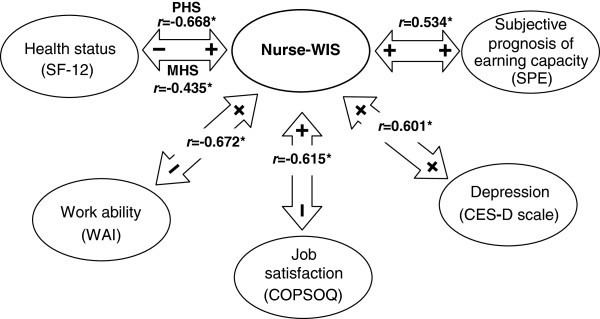
**Diagram showing correlations for testing construct validity (n = 396).***r* = Spearman correlation PHS = Physical Health Subscale MHS = Mental Health Subscale *This correlation is significant at the 0.001 level (bilateral).

All hypotheses for testing construct validity can be confirmed (Figure 1). The physical health (PHS) and the mental health subscale (MHS) of the SF-12 showed significant negative correlations with Nurse-WIS. Since the coefficient reflects the degree to which such relationships exist, the coefficient of the PHS (r = −0.668) indicates good validity and the MHS (r = −0.435) at least moderate validity. As regards the Work Ability Index (WAI) and job satisfaction (COPSOQ), there are significant correlations that suggest high construct validity. As the risk according to the Nurse-WIS increases, work ability (*r* = −0.672) and job satisfaction (*r* = −0.615) decline. The likelihood of depressive symptoms (*r* = 0.601) increases, so high validity is achieved here, too. As the risk according to the Nurse-WIS increases, the subjective prognosis of work capacity (SPE) deteriorates. Here, with *r* = 0.534, moderate validity is achieved.

### Concurrent and discriminant criterion validity

The assumptions and hypotheses for testing concurrent validity can be confirmed (Table 3). Persons with an musculoskeletal disorder (MSD) more frequently run an increased risk according to the Nurse-WIS (40.7%) than persons without an MSD (19.4%). Likewise, persons affected by a psychological impairment to well-being more often have an increased risk (44.0% versus 22.4%) according to the Nurse-WIS. As expected, no correlations appear between the risk according to the Nurse-WIS and degenerative diseases/diseases of the lower limbs or other illnesses (e.g. acute respiratory illnesses). However, there are significant differences in the case of age and length of service. Of the study participants aged between 17 and 35, 12.0% have an increased risk, while at the age of 36 to 45 and 46 to 55 the figure is around 27%, and in those over 55 years old it is 50.0%. Moreover, the proportion of persons with an increased risk according to the Nurse-WIS rises significantly with length of service. In the final binary logistic regression model, the age variable was still included and so age has an influence on risk according to the Nurse-WIS. Accordingly, study participants in the 36–45 age group (OR 2.9; 95% CI 1.47–5.63) and the 46–55 age group (OR 2.5; 95% CI 1.27–4.90) are around three times more likely to have an increased risk according to the Nurse-WIS than those aged under 35. In the case of those over the age of 55, the likelihood of having an increased risk is around seven times higher (OR 6.7; 96% CI 2.88–15.54). After controlling for age, the length of service no longer has any influence. However, the final model also includes the test criteria for criterion validity. Persons with MSD (OR 2.7; 95% CI 1.59–4.89) and those with a psychological impairment to well-being (OR 2.9; 95% CI 1.24–6.92) are around three times more likely to have an increased risk according to the Nurse-WIS.

**Table 3:** Distribution of the study population among Nurse-WIS risk categories and results of the final logistic regression model for testing discriminant validity

**Variables**	**Nurse-WIS**			
	**Low/moderate risk**	**Heightened risk**	p value*	**Final model OR (95% CI)**
	**76.3%**	**23.7%**		
	**(n = 302)**	**(n = 94)**		
**Gender**				
Female	74.6% (244)	25.4% (83)		
Male	84.1% (58)	15.9% (11)	0.094	-
**Age**				
≤ 35 years	88.0% (132)	12.0% (18)		**1**
36 to 45 years	72.6% (77)	27.4% (29)		**2.9 (1.47–5.63)**
46 to 55 years	72.1% (75)	27.9% (29)		**2.5 (1.27–4.90)**
> 55 years	50.0% (18)	50.0% (18)	<**0.001**	**6.7 (2.88–15.54)**
**Grew up in**				
Germany	77.3% (265)	22.7% (78)		
Other countries	69.8% (37)	30.2% (16)	0.236	-
**Education**				
Lower secondary, elementary school certificate	72.6% (82)	27.4% (31)		
Secondary school certificate	78.7% (166)	21.3% (45)		
High school/university entrance certificate	75.0% (54)	25.0% (18)	0.451	-
**Vocational training**				
Qualified geriatric nurse or nurse	74.3% (182)	25.7% (63)		
Geriatric care or nursing assistant	75.5% (71)	24.5% (23)		
Employee without nursing training^1^	86.0% (49)	14.0% (8)	0.172	
**Length of service**				
0–10 years	84.7% (149)	15.3% (27)		
11–20 years	71.9% (87)	28.1% (34)		
21–30 years	67.2% (39)	32.8% (19)		
More than 30 years	65.9% (27)	34.1% (14)	**0.004**	-
**Scope of employment**				
Full time (≥ 35 hours a week)	77.7% (212)	22.3% (61)		
Part time (15–34 hours a week	72.4% (84)	27.6% (32)		
Part time (< 15 hours a week)	85.7% (6)	14.3% (1)	0.452	-
**Working hours**				
Rotating shifts excluding nights	77.2% (173)	22.8% (51)		
Rotating shifts including nights	72.1% (75)	27.9% (29)		
Day duty, always at the same times	79.5% (31)	20.5% (8)		
Only night work	79.3% (23)	20.7% (6)	0.688	-
**Musculoskeletal disorders**				
No	80.6% (254)	19.4% (61)		**1**
Yes	59.3% (48)	40.7% (33)	<**0.001**	**2.7 (1.59–4.89)**
**Psychological impairments to well-being**				
No	77.6% (288)	22.4% (83)		**1**
Yes	56.0% (14)	44.0% (11)	<**0.001**	**2.9 (1.24–6.92)**
**Degenerative disease/disease of the lower limbs**				
No	76.9% (287)	23.6% (88)		
Yes	73.9% (17)	26.1% (6)	0.738	-
**Other illnesses**				
No	75.9% (167)	24.1% (53)		
Yes	77.8% (137)	22.2% (39)	0.651	-

^1^and trainees, individuals doing civilian national service and persons undertaking a voluntary year of social service.

*Pearson’s chi-square.

*OR* = Odds Ratio.

95% *CI* = 95% confidence interval.

## Discussion

This is the first study to have translated the original English version of the Nurse-WIS and to have validated this version. The psychometric characteristics of the Nurse-WIS were tested for a collective of 396 nursing staff from nursing homes. Reliability and validity were tested by various methods, and initially all items achieved acceptable degrees of difficulty. However, according to the item discrimination two items were unsuitable, so the German version of the scale comprises a total of 28 items. According to Cronbach’s, good measuring accuracy is achieved for the scale of 28 items. That means that the items on the scale are suitable for recording the construct.

In the study by Gilworth et al. [38], it was also shown that the English version of the Nurse-WIS has good face validity for registered nurses and health care assistants, and meets the measurement requirements as defined by modern psychometric theory.

Since the German version of the scale corresponds to the hypotheses formed from theory and empirical research, a high degree of construct validity can be assumed. As the risk according to the Nurse-WIS increases, health-related quality of life, the ability to work and job satisfaction decline. Simultaneously, the probability of depressive symptoms increases and the subjective prognosis of work capacity deteriorates. There are good values for concurrent criterion validity, too, since persons with a increased risk according to the Nurse-WIS more often have a musculoskeletal disorder (MSD) or a psychological impairment to well-being. The scale therefore records what it is meant to record. However, in addition to this correlation, age is an influencing factor. Older persons are more likely to have an increased risk according to the Nurse-WIS. 12.0% of those under 35 have an increased risk, while the figure for those aged over 55 is 50%. This relationship seems plausible since the Nurse-WIS is designed to record work instability primarily due to symptoms of an MSD, and MSDs occur more frequently with advancing age [7,8,65-68]. A study by Kromark *et al*. [6] likewise showed that, at 56%, the prevalence of back complaints among health care workers aged over 50 is higher than among their younger colleagues (37%). It was also found that with increasing age, the work capacity of health care and geriatric care workers declines [5,6]. Moreover, the Nurse-WIS remains within the final binary logistic regression model, i.e. regardless of age, the Nurse-WIS shows a strong correlation with the presence of an MSD or a psychological impairment to well-being.

In order to ascertain the validity of the original English version of the Nurse-WIS, Gilworth *et al*. [38] arranged for some of the study participants (n = 27) to be examined individually by an occupational therapist. The occupational therapist used a standard procedure for ascertaining the status of work instability and the result was compared to the result of the Nurse-WIS. The Nurse-WIS was found to have 75% sensitivity and 100% specificity. In the present study, no values for sensitivity and specificity have been ascertained so far, as this is to be done with the help of data from the follow-up survey. The data on sick leave was taken from the questionnaire as stated by the study participants themselves. An individual assessment of a study participant by an occupational therapist, as used by Gilworth *et al*. [38] may be more reliable. However, Gilworth *et al*. [38] did not examine whether there was actually an absence due to sickness, long-term sick leave or a pension for reduced work capacity at a later date. Therefore, there was no prospective study. This prospective study was carried out for the first time in the follow-up study of the cohort of nursing staff from nursing homes presented here. The data from this survey is currently being analysed and prepared for publication.

### Using the Nurse-WIS to maintain the work ability of health care workers

The Nurse-WIS seems suitable for identifying health care workers at risk and for designing offers of prevention efficiently so as to maintain the work ability of health care staff. The proportion of health care workers with an increased risk according to the Nurse-WIS was 23.7%. This proportion, with a conspicuous result on the Nurse-WIS, appears relatively high.

Other studies have not so far examined work instability among health care workers. Taking the 30%–60% prevalence of back complaints among staff [21-23] as a comparison, the proportion of health care workers with an increased risk according to the Nurse-WIS does seem plausible, however.

Nonetheless, considering that according to the Nurse-WIS result one would like to facilitate preventive action for one in two health care workers aged over 55, the proportion of 50% seems very high. Consequently, before the Nurse-WIS can be used as an instrument for managing health promotion, prevention or rehabilitation measures, the predictive values of the scale as well as its sensitivity and specificity should be ascertained.

### Special features of the study design and representativeness of the sample

Study participants were recruited via geriatric nursing homes. The study documents and questionnaire were displayed for nursing staff at their workplace. So that a sufficient number of study documents were sent to the homes, they informed us of the number of health care workers employed. The response rate was 23.1%. However, we suspect that some nursing homes ordered more questionnaires than were actually needed. A number of homes gave an estimated, rounded figure of the number of study documents needed (e.g. ‘Send us about 100 questionnaires’) since the care managers in charge are often unable to say off the cuff the exact number of health care workers currently employed. Often, they gave the total number of employees (including domestic workers, cleaners, etc.). It is also likely that not all the health care workers employed were present (e.g. due to sickness, holidays, pregnancy, parental leave) when the study documents were displayed, although they remained on display for several weeks. Consequently, the response rate should be regarded as an approximate figure and can be assumed to be somewhat underestimated.

However, because of the low response rate one might think there are alternative methods to assess data from nursing staff working in geriatric nursing homes, for example telephone or face-to-face interviews or electronic questionnaires (e.g. via email or website). We considered that telephone or face-to-face interviews would not be possible, because nursing staff in nursing homes will not have the time during their shift to answer the questions on the telephone or personally. And since there is no nurses association in Germany, which could have given support to announce the study and to provide an electronic questionnaire on a website, we decided to choose the option of paper questionnaires.

The proportion of health care workers with an increased risk according to the Nurse-WIS was 23.7%. Under the focus of the low response rate this proportion appears relatively high and one can assume that there is the possibility of a selective sample. But with more than 80% of female study participants the sex distribution, just as the age distribution in the present study is comparable with the distribution in geriatric care in Germany [69].

Approximately 20% said they had taken sick leave in the previous 12 months because of a musculoskeletal disorder (MSD). Furthermore, MSDs (following other illnesses such as acute respiratory or gastro-intestinal illnesses) were the most frequent reason for absence due to sickness. Some studies have found a somewhat higher prevalence rate (30% to 60%) of lower back problems among health care workers [19,22,23,70]. However, these studies do not record sick leave due to MSDs, but only MSD symptoms such as back pain or neck pain. Pain, however, does not necessarily lead to health care workers staying away from work and therefore being on sick leave. Some of these prevalence rates were also ascertained during other observation periods. In Videman *et al*. [23], for example, participants were studied over a period of five years. Back pain is likely to occur more often during a period of five years than during a 12-month observation period.

The rate of impairments of psychological well-being was approximately 6%. The literature also makes it clear that health care workers are affected by burnout and psychological impairments [24-29,71]. However, due to different conceptualisations of burnout and the absence of standard survey instruments in the studies, it is not yet possible to make a precise statement about the prevalence of burnout in geriatric care [71].

The overall conclusion is that the sample in the present study is comparable with other studies and that one can therefore assume a good degree of representativeness.

Summing up, one can say that the Nurse-WIS has shown itself to be a promising instrument with good psychometric properties. So far, the Nurse-WIS has been tested on a sample of nurses for the elderly in nursing homes, but the extent to which the Nurse-WIS is generally applicable requires further testing, for example among registered nurses or health care workers in hospitals or in outpatient care. There are other more general scales (e.g. WAI, SF-12) with good psychometric properties, but these scales often focus on health-related quality of life or on disability and function. Currently, as far as we are aware, the Nurse-WIS is the first occupation-specific scale to focus on health care workers experiencing work instability. And since there is evidence that early intervention is more effective, the identification of work instability in health care workers might be very helpful for ensuring that they have rapid access to these intervenient measures.

## Conclusion

The Nurse-WIS shows good reliability and validity, and one can assume good measuring accuracy. It therefore seems suitable for recording work instability. Moreover, the Nurse-WIS is a short, easy-to-use and low-cost instrument that seems suitable for practical use and for application in research and evaluation.

The background to the present study is the central task of countering the anticipated shortage of health care workers as the result of demographic change. Research, too, has confirmed that early prevention and health-promotion measures are effective in preventing the chronification of diseases and premature retirement [34-37,72]. Until now, however, there has been a lack of screening instruments for identifying health care workers at risk and for offering efficient and targeted prevention measures. That is why we validated the Nurse-Work Instability Scale (Nurse-WIS). This study, along with the study on the development of the Nurse-WIS [38], is the only validation study to date. However, the findings of the follow-up study are still pending. If the follow-up should also show the Nurse-WIS to be a reliable screening instrument with good predictive validity, the Nurse-WIS could help in taking early, targeted, suitable action to prevent or minimize sickness absence and potentially prevent loss of health care workers from the workforce through long-term sickness absence and early retirement.

## Abbreviations

Nurse-WIS: Nurse-work instability scale; MSD: Musculoskeletal disorders; WAI: Work ability index; SF-12: Short form health survey; SPE Scale: Subjective prognosis of work capacity; COPSOQ: Job satisfaction scale from the German version of the Copenhagen Psychosocial Questionnaire; CES-D-Scale: German version of the Center for Epidemiological Studies Depression Scale; BGW: German Institution for Statutory Accident Insurance and Prevention in the Health and Welfare Services; CVcare: Competence Centre for Epidemiology and Health Services Research in Nursing.

## Competing interests

The authors declare that they have no competing interests.

## Authors’ contributions

AB helped to develop the Questionnaire and participated in the Data collection. MH participated in the design of the study, performed the statistical analysis and drafted the manuscript. AN conceived of the study, and participated in its design and coordination and helped to draft the manuscript. All authors read and approved the final manuscript.
